# Blood Flukes and Arterial Damage: A Review of Aneurysm Cases in Patients with Schistosomiasis

**DOI:** 10.1155/2022/6483819

**Published:** 2022-12-03

**Authors:** Valeria Silvestri, Vivian Mushi, Mwanahawa Idavas Mshana, Witness M. Bonaventura, Nyanda C. Justine, Deodatus Sabas, Billy Ngasala

**Affiliations:** ^1^Department of Parasitology and Medical Entomology, Muhimbili University of Health and Allied Sciences, P.O. Box 65011, Dar es Salaam, Tanzania; ^2^Department of Zoology and Wildlife Conservation, College of Natural and Applied Sciences, University of Dar Es Salaam, Dar es Salaam, Tanzania; ^3^Kilimanjaro Christian Medical University, Moshi, Tanzania; ^4^Directorate of Library Service, Muhimbili University of Health and Allied Sciences, P.O. Box 65001, Dar es Salaam, Tanzania

## Abstract

**Introduction:**

Schistosomiasis, caused by trematode worms of the genus *Schistosoma*, has organ-specific morbidity due to host's inflammatory response to the oviposition of parasite eggs in vessels and organs. Damage to the cardiovascular system, including aneurysms, has been described in patients. *Aims and Methods*. Aims of the review of case reports and series published in literature were to describe the occurrence of aneurysm in patients with schistosomiasis. *Investigation Outcomes*. A total of 13 cases (seven males and six females) with a mean age of 41.3 ± 14.9 years were included. Aneurysm occurred in patients with active or previous infection. In more than half of the cases, an intestinal or hepato-splenic involvement was reported, followed by pulmonary schistosomiasis and urinary or testicular involvement. The most frequently involved arterial district was the pulmonary artery. Immunomodulation and thrombophilia were featuring challenging surgery.

**Conclusions:**

More studies are needed to shed light on the vascular complications of schistosomiasis, to ascertain the true burden of aneurysms in patients with schistosomiasis, to establish the pathophysiology of vessel damage and aneurysm formation, and to assess if there is an association between schistosomiasis and aneurysm formation in line with WHO 2021–2030 NTD Roadmap.

## 1. Introduction

Schistosomiasis is an infection caused by trematode worms of the genus *Schistosoma*, which have different geographical distribution [1, 2] as follows: *Schistosoma haematobium* is endemic in sub-Saharan Africa and Middle East, *S. mansoni* is endemic in sub-Saharan Africa, South America, and Caribbean's, and *S. japonicum* is endemic in People's Republic of China, Philippines, and Indonesia [1].

Globally, over 250 million people are infected worldwide, 201.5 million of them living in Africa, particularly sub-Saharan Africa [3].

Infection in humans occurs by contact with fresh water contaminated by free-swimming cercariae that penetrate the intact human skin and enter venous and lymphatic small vessels. A symptomatic acute stage occurs two weeks to three months after exposure [1], and it is caused by systemic hypersensitivity reactions and formation of immune complexes in response to antigens released during schistosomula migration [1]. In the following stage of infection, oviposition by mature adult worms occurs [1]. Eggs reach the lumen of intestine and genitourinary tract via the characteristic spines or through the induction of an inflammatory granulomatous response, triggered by antigenic glycoproteins. In the chronic stages of the disease, eggs that are not excreted can be trapped into granulomas in tissues and can lead to different organ damage according to the infecting *Schistosoma spp* and its specific preferred sites (intestinal, hepato-splenic, and genito-urinary, but also ectopic sites such as spleen, lungs, skin, and central nervous system) [1, 4, 5].

Aneurysms are diagnosed when an increase in abdominal aortic diameter ≥30 mm on ultrasonography or CT imaging is observed [6]. Abdominal aortic aneurysm prevalence in general population is 1-2%. The main complication of aneurysms is rupture; its risk amounts to 30% a year, and it is diameter dependent. Aortic rupture leads to 150,000–200,000 death each year worldwide [6]. Aneurysms have been described in patients with other diseases of parasitological interest, such as amoebiasis [7] or hydatidosis [8, 9]. Cardiovascular system damage has been reported also in *Schistosoma* infection in the form of cerebrovascular events leading to stroke [10] or aneurysm involvement of several arterial districts [11–19].

## 2. Aims and Methods

This study aimed to describe, through a review of cases in the literature, the occurrence of aneurysm lesions in patients with schistosomiasis.

### 2.1. Search Strategy and Inclusion and Exclusion Criteria

**Figure 1:**
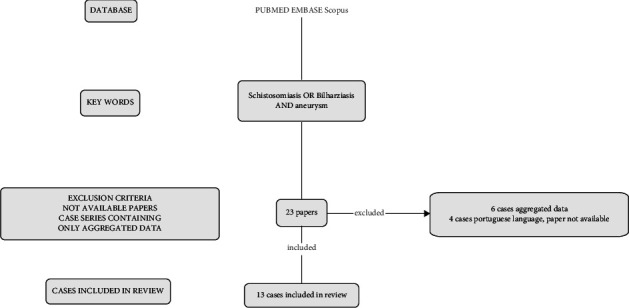
Review process flow-chart.

PubMed, Scopus, and Embase were searched, using “Schistosomiasis” OR “Bilharziasis” AND “aneurysm” as keywords. A filter for the English language was applied. Only cases with available full text and data on age, sex, and aneurysm localization were included. We excluded reports that provided aggregated data. A flow chart on the research strategy has been provided in Figure 1.

### 2.2. Quality Assessment

Case reports are assessed by making an overall judgement about methodological quality defining the availability of the whole investigator's experience such as if authors provided clear information on exposure to *Schistosoma*; if a clear report of patient's outcome, differential diagnosis, description of challenges; dose-response effect description for treatment, sufficient length of follow-up, details to allow inference-making were provided [20].

### 2.3. Data Analysis

The data were analyzed using the computer software JASP, version 0.14.1. Descriptive statistics (mean, standard deviation, median, and 25th–75th percentile) were used to present continuous variables. Percentages and frequencies were generated for categorical variables. The association statistical test was not performed, given the dis-homogeneity of reports.

## 3. Investigation Outcomes

### 3.1. Selection Process

A total of 23 cases were retrieved thorough review of the literature. Six cases from a case series of pulmonary aneurysms were excluded because data were reported as aggregated [21]. Four spleen aneurysm cases were excluded because unavailability of the article, not even by contact of the first author, additionally to the language filter applied. The flow chart of the review process has been provided in Figure 1. The median value of Murad case quality score was 2/8 with a minimum of 2 and a maximum of 7.

### 3.2. Summary of Cases

A total of thirteen cases were included in the review. Cases are summarised in Tables 1 and 2, and findings are summarised in Tables 3 and 4.

### 3.3. Stage of Schistosomiasis at Aneurysm Diagnosis

Vascular lesions were diagnosed at any stage of infestation. In some cases, they occurred in patients with active *Schistosoma* infection. In these cases, the diagnosis of *Schistosoma* infection was reached by serological investigations [22], stool sample assessment, rectal biopsy [19], and histology of lung [17] or of liver and testis [23].

Aneurysm occurred also in patients with known chronic schistosomiasis [12], in an advanced stage with clinically manifest complications, such as oesophageal varices [14], hepato-splenic schistosomiasis [16, 24, 25], in one case waiting for liver transplant [16] and in pulmonary schistosomiasis [12].

Finally, cases were described in patients that had lived in endemic areas [12, 15]. Diagnosis of arterial lesion also occurred in these cases of patients that were negative to parasitological investigations (Kato-Katz and rectal biopsy) [15], many years after leaving the endemic country [15]. Interestingly, other chronic changes related to the portal system in patients with the neglected tropical disease have been reported in refugees, as in the case of tropical splenomegaly, which may persist also months after leaving an endemic country and which suggest a possible underlying persistent infection and immune response as a cause of chronic arterial damage [26] (Tables 3 and 4).

### 3.4. Age of Patients at Diagnosis

When we analysed the age at which patients were diagnosed with an aneurysm, we observed that young patients can be affected. The youngest patient included in our review was 18-years old, and overall, five patients were below 40 years [13, 18, 19, 22, 27]. This differs from the usual age at diagnosis of atherosclerotic aneurysms and suggests the need for an increased clinical suspicion of aneurysm as differential diagnosis when assessing patients of young age with compatible clinical presentation in endemic areas (Tables 3 and 4).

### 3.5. Pathophysiology of Arterial Damage in Schistosomiasis

Vascular complications in schistosomiasis can be caused by several pathophysiological mechanisms. Haemodynamic impairment is secondary to the chronic granulomatous inflammation of embolized eggs reaching the arteriolar districts of perfused organs. The hemodynamic mechanism is well described by Zaky in 1962 in a case series of pulmonary aneurysms in schistosomiasis patients. Schistosomiasis is considered one of the main causes of pulmonary artery aneurysms [13]. According to his report, aneurysms are a late result of the local weakening of the vessel wall associated with a rise of pressure due to repeated implantation of *Schistosoma* ova [13]. This leads to increased share stress due to the onset of pulmonary hypertension, right ventricular dilatation, and cor pulmonale [17]. *Schistosome* eggs embolization may cause direct damage to the walls of arteries through additional mechanisms, including vasa vasorum obliterative endarteritis, direct endothelium damage, or inducing atheromatic degeneration [19]. Vanker describes these mechanisms. Eggs of *Schistosoma mansoni* spread from the left upper lobe of the lung to the adjacent pleura and the aortic thoracic sheath, causing endarteritis obliterans of its vasa vasorum and aneurysm [19]. Interestingly, this physio-pathological mechanism is shared by other infectious diseases associated with thoracic aortic aneurysms, such as tertiary syphilis [28].

Visceral aneurysms may also share physio-pathological mechanisms behind pulmonary lesions through hemodynamic impairment due to hepatic fibrosis and portal hypertension or as direct damage to the artery wall due to local inflammation [14, 15]. In the monocentric experience by Lambertucci et al., which included 82 patients with hepatosplenic schistosomiasis, only one case (1.2%) of visceral aneurysm (splenic artery aneurysm and intrahepatic shunt) was observed, a finding that was at first missed during ultrasound examination [14].

**Figure 2:**
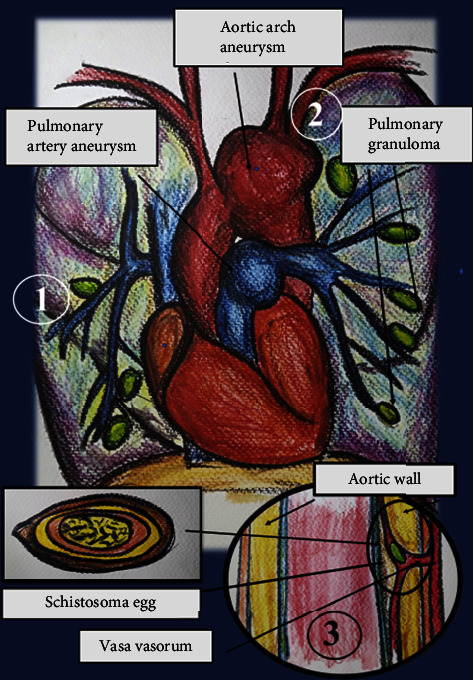
Aneurysms and schistosomiasis. Physiopathology behind aortic aneurysm and pulmonary artery aneurysm formation. (1) *Schistosoma* eggs may seed pulmonary arteries, leading to lung inflammatory reaction and granuloma formation. Direct damage to vessels may occur as a result of inflammation of tissues surrounding vessels. (2) Pulmonary inflammation due to *Schistosoma* eggs seeding of pulmonary arteries may lead to pulmonary hypertension. Damage to vessels may be due to hemodynamic changes. (3) *Schistosoma* eggs may seed major vessels wall through vasa vasorum, causing cystic medial necrosis and loss of wall integrity.

**Figure 3:**
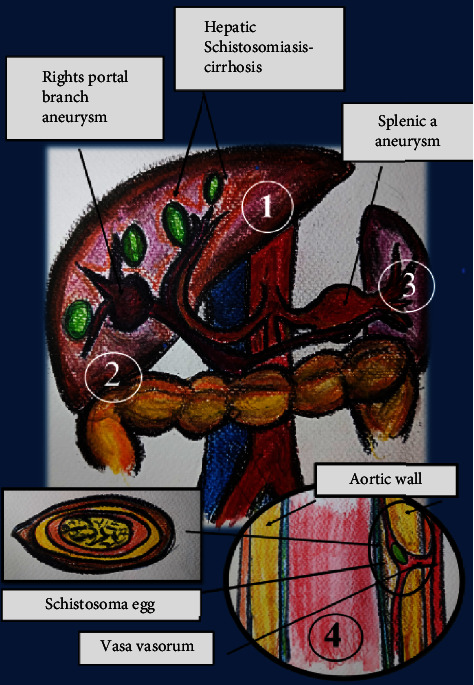
Aneurysms and schistosomiasis. Physiopathology behind portal branch aneurysm and splenic artery aneurysm formation. (1) Seeding of hepatic vessels by *Schistosoma* eggs may lead to inflammatory reaction in hepatic parenchyma, fibrosis, and direct damage to vessels. (2) Inflammatory reaction and portal fibrosis due to *Schistosoma* oviposition in hepatic vessels may lead to portal hypertension, which may induce aneurysms by hemodynamic mechanisms in visceral vessels. (3) Splenic artery aneurysm may be secondary to portal hypertension. (4) Schistosoma eggs may seed arterial wall through vasa vasorum, inducing direct damage and predisposing to aneurysm formation. Splenic a. = splenic artery. Figures 2 and 3 are original art work by the author V.S.

A pictorial view of the pathophysiology leading to pulmonary artery and visceral arteries is shown in Figures 2 and 3.

### 3.6. Histological Findings

Where available, histological findings supported the aforementioned physiopathological mechanisms. A histological report was available for three of the included cases. In case 2 reported by Vanker, of an aortic arch pseudoaneurysm, *Schistosoma* eggs were found on histological examination in pulmonary parenchyma, pleura, and arterioles, where they appear surrounded by a granulomatous reaction [19].

In case 9, of a thoraco-abdominal aortic aneurysm, histology revealed eggs in liver and large bowl and calcified eggs in lung and testis [23].

In case 3, of a right portal branch aneurysm, splenic, and liver fibrosis, destruction of portal vein branches was observed. In this case though, histology failed to detect *Schistosoma* eggs in the samples [15].

### 3.7. Immunomodulation and Infectious Complications

*Schistosomes*, specifically in the egg stage, have strong immunomodulatory effects on the immune system of their hosts and impair those immune responses necessary to combat other pathogens and to develop a protective antibody response [29]. Immune impairment could favour the occurrence of vascular damage in affected patients, as described for other disease of infectious interest [30]. This can be especially true in patients with pre-existing cardiovascular conditions or a history of previous vascular surgery as in case 11, reported by Romero de Oliveira, of an aortic rupture occurring on an aortic graft infection due to *Porphyromonas pogonae* [24].

### 3.8. Schistosomiasis and Thrombophilia

Thrombophilia has been reported as a complication in schistosomiasis patients with aneurysms. In case 8, by Abdelnaby et al., a pulmonary artery aneurysm was complicated by atrial mural thrombus [12], and in case 7, by Abo-Salem and Ramadan, a massive pulmonary embolism complicated a pulmonary artery aneurysm after the patient's refusal of surgical treatment [17]. Also, in case 3, reported by Mucenic, the postoperatory for splenectomy in a patient with hepatic artery aneurysm was complicated by partial spleen vein thrombosis. Inflammation-induced thrombophilia, associated with schistosomiasis, has been suggested as the feature favouring the formation of thrombus in the aneurysm lumen. Secondary thrombophilia should thus be considered an additional cardiovascular risk factor in this setting [12].

Pathophysiological mechanisms that lead to aneurysm in patients with schistosomiasis are described in Figures 2 and 3.

### 3.9. Considerations on Surgical Treatment

Different kinds of approaches have been described according to the location of the aneurysm.

Aneurysmectomy and direct suture have been used to treat an aortic arch pseudoaneurysm by D. A. Athanazio and P. R. F. Athanazio [23]. Nephrectomy, aneurysmectomy, and renal reimplantation have been successfully described in a renal artery aneurysm [25]. Splenectomy has been considered in a portal branch aneurysm associated with portal hypertension. By reducing portal hypertension, splenectomy favoured the reduction of aneurysm size to 1.4 cm at 4 year follow up [15], suggesting that the excessive venous inflow from the splenic vein was contributing to portal hypertension and vessel dilatation [15].

Aneurysm lesions may be prone to reduction in size when the haemodynamic condition that favours their development is treated. Recently, transjugular intrahepatic portosystemic shunt has been suggested as a reversible and a far less invasive alternative to surgery in patients with portal hypertension, including those due to *Schistosoma* infection, preserving spleen and its immunologic function [31]. The limited availability in an endemic setting, the high cost, and marginal indications in guidelines challenge the assessment of the safety and effectiveness of this technique in this specific setting [32] (Tables 3 and 4).

### 3.10. Outcome

Some specific features of aneurysms in patients with schistosomiasis have to be considered when it comes to giving surgical indication. Aneurysms wall local weakening due to the rise of pressure and to the implantation of *Schistosoma* ova has been described [21]. These features could make aneurysms lesions in this context highly prone to rupture [16]. In case 5, exitus was reported while waiting for surgery for a pulmonary artery aneurysm, due to rupture and cardiac tamponade [16]. Rupture also occurred in a hepatic aneurysm in a patient with hepatic abscess, which occurred notwithstanding the pharmacological management with praziquantel [22]. Thrombophilia related complications have to be considered when planning surgery; partial portal vein thrombosis complicated splenectomy in the case by Mucenic et al. [15] and exitus occurred in case 7 because of massive embolization from a pulmonary artery aneurysm, notwithstanding anticoagulant treatment [17].

Finally, general conditions can be impaired by the high prevalence of pulmonary or portal hypertension but will also be secondary to effects of immune-modulation [24], favouring overlap of infections, as in the case of hepatic aneurysm in a patient with schistosomiasis and *Fasciola hepatica* infection [16] and occurrence of sepsis complications, as in the aortic graft infection in a patient with chronic schistosomiasis and history of aortic repair for type A aortic dissection [24]. Additionally, clinical features can be exacerbated by malnutrition, which can be significantly prevalent in endemic areas [21] (Tables 3 and 4).

## 4. Conclusions and Recommendations

Our review of available literature (limited to case reports and case series) has described the co-occurrence of aneurysm lesions in patients with schistosomiasis, suggesting the need to ascertain the true burden of aneurysms in patients with schistosomiasis, to establish the pathophysiology of vessel damage and aneurysm formation and to assess if there is an association between schistosomiasis and aneurysm formation. These studies will be in line with the newly-launched revised WHO 2021–2030 NTD Roadmap, towards the elimination of morbidity related to Neglected Tropical Diseases in all endemic countries by 2030 [2, 10].

## Figures and Tables

**Table 1:** Summary of cases.

*N*	Author, year	Age, sex	Geographic region	Schistosoma species	Comorbidities and c.v. risk factors in anamnesis	Schistosomiasis details	Clinical presentation	Vascular findings	Pharmacological treatment of schistosomiasis
1	Salah, 1997	27, M	Egypt	n.a.	Smoke	Previous urinary andintestinal schistosomiasis, normal lung parenchima	Cardio-vocal Ortner's syndrome; exertional dyspnoea (2 months), pulmonary hypertension	Pulmonary artery-aneurysmand schistosomal, cor pulmonale	Previous treatment with praziquantel for intestinal and urinary schistosomiasis
2	Vanker, 1986	19, F	South-Africa	*Schistosoma mansoni* (stool and rectal mucosa)	None	Pulmonary shistosomiasis	Haemoptysis; left chest pain, hoarseness, absent left brachial pulse (1 month), haematuria	Aortic arch pseudoaneurysm (7 x 2 cm)	Not reported
3	Mucenic, 2002	45, M	Lived for 17 years in *Schistosoma* endemic area	Negative (stool and rectal biopsy)	No other	Previous hepatosplenic schistosomiasis (hematemesis, enterorrhagia, oesophageal varices)	Abdominal continuous pain (left hypochondrium)	Right portal branch aneurysm (5 x 4 x 4 cm)	No previous treatment with praziquantel
4	Lambertucci, 2010	66, F	Brazil	n.a.	No other	Chronic hepato-splenic schistosomiasis (esophago-gastric varices; hematemesis)	Routine assessment for hepato-splenic schistosomiasis	Saccular aneurysm of the splenic artery; intrahepatic shunt between right portal branch and right hepatic vein	Beta-blockers as gastroenteric haemorrhage prophylaxis
5	Piveta, 2012	41, F	Brazil	n.a.	Alcoholic hepatitis waiting for liver transplant	Hepato-splenic schistosomiasis	Intermittent chest pain; pulmonary hypertension; intrapulmonary shunt	Pulmonary a. aneurysm (8.3 cm)	Not reported
6	Genzini, 2014	48, M	Brazil	n.a.	Hypertension, thrombo-cytopenia	Advanced hepatosplenic schistosomiasis	Right lumbar pain	Right renal artery aneurysm 2.5 cm	Not reported
7	Ramadan, 2015	55, M	Lower-Egypt	*Schistosoma mansoni* (lung biopsy)	Ex-smoker	History of intestinal and hepato-splenic schistosomiasis. Pulmonary granuloma, positive serology *S. mansoni*	Dyspnoea (one month); fever (2 months) cough and mucoid sputum (10 days) left atrial compression; pleural effusion	Right pulmonary artery aneurysm (17 x 11 cm)	Previous treatment for intestinal schistosomiasis (20 years previously). Anticoagulation
8	Abdelnaby, 2018	50, F	Egypt	n.a.	n.a.	Bilharziasis since early childhood	Chest pain (recurrent); exertional dyspnoea	Right and left pulmonary a. aneurysm (6.5 cm) and atrial mural thrombus	Anticoagulation
9	Athanazio, 2018	n.a., M	Brazil	*Schistosoma mansoni* (lung, testis, liver, and large bowl)	n.a.	Testicular, intestinal and lung schistosomiasis	Spontaneous aorto-cutaneous fistula	Thoraco-abdominal aortic aneurysm; spontaneous aorto-cutaneous fistula	n.a.
10	Gavilanes, 2018	38, M	Brazil	n.a.	n.a.	Chronic schistosomiasis, pulmonary artery hypertension	Palpitations; dyspnoea; exertional syncope	Giant pulmonary artery aneurysm; aorta and left coronary artery compression	n.a.
11	De Oliveira, 2019	48, M	Brazil	n.a.	Type A aortic dissection ascending aorta substitution (dacron); biological valve	Hepato-splenic schistosomiasis	Fever *Porphyromonas pogonae* sepsis	Aortic graft infection and aortic rupture	Antibiotics for associated bacteria
12	Dyer, 2020	18, F	Australia	*Schistosoma* Ag EIA	n.a	Serology positive for schistosomiasis	Fever; right upper quadrant pain; liver abscess *Fasciola hepatica* infestation	Hepatic artery pseudoaneurysm; acute bleeding from the ampulla of Vater	Praziquantel
13	Abdelnaby, 2020	38, F	Egypt	n.a.		History of bilharzias	Dyspnoea (5 years) and hoarseness (1 year)	Pulmonary artery aneurysm (PAA) with dilatation of both branches and hoarseness	Anticoagulation

Anagraphic details, geographic region of patient's origin/residency, schistosomiasis details, clinical presentation, vascular findings, and pharmacological management are included. M = male; F = female; Ag EIA = antigen enzyme-linked immunosorbent essay; n.a = not available; c.v. = cardiovascular. The most frequently involved arterial district was the pulmonary artery, followed by aortic lesions which included one recurrent lesion on previous aortic surgery and abdominal visceral vessels aneurysms (specifically in splenic, hepatic artery, right portal branch, and renal artery).

**Table 2:** Surgical treatment and outcome of patients.

*N*	Author, year	Age, sex	Vascular findings	Surgical treatment	Outcome
2	Vanker, 1986	19, F	Aortic arch pseudoaneurysm (7 x 2 cm)	Aneurysmectomy and direct suture	Unknown
3	Mucenic, 2002	45, M	Right portal branch aneurysm (5 x 4 x 4 cm)	Splenectomy	Partial portal vein thrombosis. Alive (reduction of aneurysm size to 1.4 cm and resolution of the vessel thrombosis on follow up)
4	Lambertucci, 2010	66, F	Saccular aneurysm of the splenic artery; intrahepatic shunt between right portal branch-and right hepatic vein	Refused surgery treated with beta-blockers	Unknown
5	Piveta, 2012	41, F	Pulmonary artery aneurysm (8.3 cm)	Exitus waiting for surgery	Exitus (aneurysm rupture and cardiac tamponade)
6	Genzini, 2014	48, M	Right renal artery aneurysm 2.5 cm	Nephrectomy, aneurysmectomy and renal reimplantation	Alive (creatinine improvement on follow-up)
7	Ramadan, 2015	55, M	Right pulmonary artery aneurysm (17 x 11 cm)	Refused surgery on anticoagulation	Exitus (massive pulmonary embolization, notwithstanding anti-coagulant therapy)
11	De Oliveira, 2019	48, M	Aortic graft infection and aortic rupture	Not-specified reintervention on previous aortic graft on antibiotic therapy	Exitus (complications of surgery on 12^th^ day post-operatory, likely due to comorbidities related to hepato-splenic schistosomiasis)
12	Dyer, 2020	18, F	Hepatic artery pseudoaneurysm; acute bleeding from the ampulla of Vater	Not specified in therapy with praziquantel and	Bleeding of aneurysm from Vater ampulla, notwithstanding praziquantel
13	Abdelnaby, 2020	38, F	Pulmonary artery aneurysm with dilatation of both branches and Ortner's syndrome	Refused surgery	Discharged on close follow-up

Anagraphical details, aneurysm description, surgical management, and outcome of patients were specified. Fatality was reported in 3/13 patients. F = female; M = male.

**Table 3:** Summary of findings.

Clinical presentation
The most frequently reported symptom at presentation was pain referred to the chest, hypochondrium, or the lumbar region, followed by dyspnoea, fever, hoarseness and Ortner's syndrome, hyper-eosinophilia, syncope, cardiogenic shock, and anaemia
Cardiovascular risk factors and comorbidities
Cardiovascular issues were the most frequent associated comorbidity, including hypertension, previous surgery for type A aortic dissection; oesophageal varices, a history of smoke and alcohol consumption were also described in some patients
Timing of schistosomiasis diagnosis and arterial damage observation
A history of previously diagnosed schistosomiasis was reported in the majority of cases or in patients with known chronic schistosomiasis, in an advanced stage with ouverte complications. Cases have been also described in patients that had lived in endemic areas previously to the vascular diagnosis even if negative to parasitological investigations and many years after leaving the endemic country
Arterial districts involved by aneurysm lesions
The most frequently involved arterial district was the pulmonary artery, followed by aortic lesions which included one recurrent lesion on previous aortic surgery and abdominal visceral vessels aneurysms (specifically in splenic, hepatic artery, right portal branch, and renal artery)
Laboratory investigations
Diagnosis of actual infection was reached by different methods, including serology, stool sample analysis, biopsy of lung, liver, or testis
Pharmacological treatment
Case 4: beta-blocker gastro-enteric haemorrhage preventive therapy in splenic artery aneurysm with intrahepatic shunt
Case 7: heparin and then anticoagulant in pulmonary artery aneurysm
Case 8: anticoagulant for pulmonary artery aneurysm complicated by atrial thrombus
Case 11: ciprofloxacin, piperacillin, and tazobactam then switched to meropenem and metronidazole for aortic graft infection and rupture on previous type A dissection surgery
Case 12: praziquantel and triclabendazole for hepatic pseudoaneurysm in hepatic abscess
Case 13: antifailure measures and anticoagulants
Surgical treatment
Case 2: aneurysmectomy and direct suture of pseudoaneurysm of aortic arch
Case 3: splenectomy for right portal branch aneurysm and portal hypertension
Case 6: nephrectomy, aneurysmectomy, and kidney reimplantation for renal artery aneurysm
Case 11: nonspecified reintervention for aortic graft infection and aortic rupture
Outcome
Case 3: portal vein thrombosis after splenectomy, resolved after 4 years follow-up. In 3 cases exitus was reported
Case 5: comorbidities did not allow surgery for pulmonary artery aneurysm and cardiac tamponade
Case 7: massive pulmonary embolism complicating a pulmonary artery aneurysm for which treatment had been refused
Case 11: heart failure in the late postoperatory period for reintervention on previous aortic graft
Case 12: hepatic artery bleeding through ampulla of Vater notwithstanding pharmacological therapy with praziquantel
Case 13: alive, refused surgery, discharged on anticoagulant and antifailure measures

The clinical presentation, cardiovascular risk factors and comorbidities, timing of schistosomiasis diagnosis and arterial damage observation, arterial districts commonly involved, the diagnostic and therapeutic management, and outcome of patients are summarised.

**Table 4:** Summary of findings with frequencies and percentages.

Anagraphic details	Mean ± SD	Range
Age	41.0 ± 14.3	18–66
Sex	***N***	%
Male	7	53.8
Female	6	46.1
Cardiovascular risk factors and comorbidities		
Cardiovascular comorbidity (Hypertension, previous type A aortic dissection, and pulmonary valve steno-insufficiency)	3	23.1
Oesophageal varices	2	15.4
Ex-smoker	2	15.4
Alcohol abuse	1	7.7
Schistosomiasis details		
Previous history of schistosomiasis	8	61.5
Actual positivity for schistosomiasis (stool, antigen test, or histology)	4	30.8
Type of schistosomiasis		
Intestinal-hepatosplenic	7	53.8
Pulmonary	5	38.5
Urinary or testicular	2	15.4
Clinical presentation		
Pain (chest, hypochondrium, and lumbar)	6	46.1
Dyspnoea	6	41.6
Fever	3	23.1
Hoarseness	3	23.1
Hyper-eosinophilia	2	15.4
Syncope/cardiogenic shock	2	15.4
Anaemia	1	7.7
Artery involved		
Pulmonary	6	41.6
Aortic arch	1	7.7
Thoraco-abdominal	1	7.7
Portal	1	7.7
Hepatic	1	7.7
Renal	1	7.7
Splenic	1	7.7
Rupture on previous aortic graft	1	7.7
Treatment		
No surgery (3 refusals, 1 exitus, 1 n.s)	5	38.5
Splenectomy	1	7.7
Nephrectomy, aneurysmectomy, and renal reimplantation	1	7.7
Aneurysmectomy (aortic arch)	1	7.7
Not-specified procedure (aortic rupture on previous graft)	1	7.7
Outcome		
Not specified	6	41.6
Alive	4	30.8
Exitus	3	23.1

Anagraphic details, cardiovascular risk factors and comorbidities, schistosomiasis details (previous history or actual positivity) and type of schistosomiasis according to the apparatus involved, clinical presentation (signs and symptoms), arterial segment involved, treatment (conservative or surgical), and outcome are summarised, providing mean and standard deviation (for age) and number of cases and percentage for other factors. SD = standard deviation.

## Data Availability

All available data have been included in the manuscript.
